# Mapping dysphagia in pediatric dystonia

**DOI:** 10.1371/journal.pone.0324140

**Published:** 2025-06-18

**Authors:** Muserrefe Nur Keles, Esra Serdaroglu

**Affiliations:** 1 Department of Physiotherapy and Rehabilitation, Gazi University Faculty of Health Sciences, Ankara, Turkiye; 2 Department of Pediatric Neurology, Gazi University Faculty of Medicine, Ankara, Turkiye; Shaheed Rajaei Cardiovascular Medical and Research Center: Rajaie Cardiovascular Medical and Research Center, IRAN, ISLAMIC REPUBLIC OF

## Abstract

**Objectives:**

Dystonia is a significant movement disorder in childhood, yet swallowing functions in this population remain largely unexplored. Dysphagia, however, can result in severe complications, including aspiration, underscoring the critical need for research in this area. This study, therefore, aimed to evaluate dysphagia in children with dystonia.

**Methods:**

Children diagnosed with dystonia as the predominant movement disorder were included. Medical histories were recorded, and Gross Motor Function Classification System (GMFCS) and Functional Oral Intake Scale (FOIS) levels were determined. Oral structure characteristics were assessed, and chewing performance was evaluated using the Turkish version of Mastication Observation and Evaluation (T-MOE) and the Karaduman Chewing Performance Scale (KCPS). Swallowing safety was screened with the Pediatric Eating Assessment Tool-10 (PEDI-EAT-10) and the 3-ounce Water Swallow Test. The Dysphagia Disorders Survey (DDS) was used to assess swallowing disorder severity, while the Dysphagia Management Staging Scale (DMSS) was applied to determine the severity level of dysphagia.

**Results:**

Twenty-five children (mean age: 11.32 ± 3.95 years) participated in the study. Of these 56% were classified as level V according to the GMFCS. Three children (12%) had a FOIS level of 4 or below. The mean T-MOE score was 15.62 ± 7.51, and 60% of the children could bite but could not chew effectively according to the KCPS. Oropharyngeal dysphagia was present in all children, with abnormal swallowing (PEDI-EAT-10 score ≥4) and increased aspiration risk (PEDI-EAT-10 score ≥13) observed in 100% and 88% of the participants, respectively. Additionally, 52.0% of the children failed the 3-ounce Water Swallow Test. The mean DDS raw score was 23.08 ± 7.70, and 68% of the children were classified as having severe or profound dysphagia based on the DMSS.

**Conclusion:**

Swallowing dysfunction was observed in almost all children with dystonia, with the majority presenting with severe dysphagia and an elevated risk of aspiration. Close monitoring of oral structures and functions, along with continuous evaluation of swallowing performance, is crucial to ensure safe oral feeding and to mitigate life-threatening complications in this population.

## Introduction

Dystonia is a movement disorder characterized by involuntary, sustained, or intermittent muscle contractions that lead to abnormal twisting and patterned postures and/or movements [1]. These symptoms typically worsen with voluntary actions, resulting in functional impairments in daily life. In pediatric cases, dystonia often coexists with other movement disorders, complicating both diagnosis and management. According to a recent meta-analysis, the global prevalence of idiopathic or inherited isolated dystonia is estimated at approximately 30.85 per 100,000 individuals [2]. However, the prevalence of dystonia in the pediatric population remains unknown. While traditionally classified by phenomenology, recent advances in neurogenetics and neuroimaging have deepened our understanding of its diverse presentations and underlying mechanisms [3].

The etiology of dystonia is heterogeneous and can be divided into genetic and acquired forms. Recent studies have identified multiple causative genes, such as *KMT2B*, *WARS2*, and *SLC18A2*, each associated with distinct clinical profiles and age of onset [4,5]. Among these, *KMT2B*-related dystonia is increasingly recognized due to its early onset and responsiveness to deep brain stimulation [6]. Acquired dystonia, including those secondary to hypoxic-ischemic injury or cerebral palsy (CP), remain common in clinical settings, but their features may be masked by coexisting spasticity or mixed movement patterns [7].

Swallowing is one of the most fundamental functions necessary for sustaining life, alongside breathing. Although this vital function may seem limited to the oral, pharyngeal, and esophageal regions, it is highly complex due to its neurophysiological, anatomical, and biomechanical connections [8]. As a result, it has both direct and indirect links with many other structures in the body. In children with dystonia, altered muscular tone and involuntary motor output can interfere with the normal biomechanics of swallowing. Additionally, recent reviews have drawn attention to non-motor symptoms in dystonia-including feeding and swallowing difficulties-that may go under recognized yet significantly impact quality of life [9].

Although there are no studies specifically examining swallowing function in children with dystonia, some research has focused on children with CP from which dystonia also has etiological overlap. In these studies, children with dyskinetic CP constituted a relatively small proportion of the sample size. Nevertheless, the studies reported impairments in especially oral and pharyngeal phases of swallowing [10–12]. Despite these findings, extrapolating results from children with cerebral palsy to those with dystonia may lead to inaccurate conclusions due to key differences in pathophysiology and clinical presentation. These distinctions are crucial for developing effective assessment and intervention strategies tailored to each patient’s condition. However, research specifically examining swallowing impairments in children with dystonia is limited, leaving a significant gap in clinical knowledge.

This study aims to address this knowledge gap by examining the clinical characteristics of swallowing dysfunction in children with dystonia, with particular focus on how etiological differences shape its presentation and management. By contributing data grounded in recent calls for personalized assessment in pediatric dystonia [13], we seek to enhance multidisciplinary care and improve outcomes for this complex patient group.

## Materials and methods

### Study population

This cross-sectional study was conducted between 17 September 2023 and 9 January 2025 at Gazi University Hospital, a leading center in Turkey for the diagnosis and management of pediatric dystonia. Patients from various cities across the country are referred to our center, where children with dystonia are evaluated and treated by a highly specialized multidisciplinary team including specialists from pediatric neurologist, pediatric medical genetics, child psychiatrists, pediatric physiotherapist, and nutritionist and dietitian. Surgical treatments are discussed and planned in the “Deep brain stimulation multidisciplinary meetings” led by movement disorders specialists from pediatric neurology, adult neurology, neurosurgery, and neuropsychology units. However, the clinical assessments in this study were conducted by a pediatric neurologist experienced in movement disorders and a physiotherapist specialized in swallowing disorders. A total of 25 children aged between 2 and 18 years were included in the study if they met the following criteria: a confirmed neurological diagnosis, such as cerebral palsy, metabolic disorders, or genetic syndromes, with dystonia as the predominant movement disorder. The exclusion criteria encompassed conditions that could interfere with the evaluation process, including active or untreated seizures and acute medical illnesses, such as mouth ulcers, tonsillitis, pharyngitis, or pneumonia. Before participation, written informed consent was obtained from all participating children’s parents or legal guardians, ensuring ethical compliance and a clear understanding of the study’s objectives.

Dystonia is a relatively rare condition, which makes it challenging to determine an optimal sample size. Given the lack of prior studies specifically addressing this population, we adopted an inclusive approach, enrolling all eligible children diagnosed with dystonia during the study period.

### Study design

The medical histories included the children’s diagnoses, neurological evaluations, prominent movement disorders, medications, and pneumonia episodes in the last year. Following this, the children were referred to a therapist specializing in swallowing disorders for a comprehensive evaluation of their swallowing function. Considering the potential stress and excitement caused by unfamiliar settings and new individuals, the evaluation environment was designed to minimize these factors. Efforts were made to create a familiar, quiet, and non-crowded space where children could feel comfortable.

Swallowing evaluations started with determining the patients’ functional oral intake levels. The assessment systematically examined the physical structure and functionality of the oral anatomy, chewing function and its efficiency, saliva frequency and severity, and tongue thrust severity. These initial evaluations were completed within approximately 15 minutes. Afterward, the 3-ounce water swallow test was administered to assess the safety of oropharyngeal swallowing, and caregivers were asked to complete the Pediatric Eating Assessment Tool-10 (PEDI-EAT-10) swallowing questionnaire. In the final phase of the swallowing evaluation, the severity of swallowing disorders was determined using the Dysphagia Disorder Survey (DDS), a specialized assessment tool that can only be administered by certified specialists. In this study, a certified therapist (M.N.K) performed all DDS assessments.

The DDS assessment was conducted using two methods. The first method involved observing and evaluating the children in a clinical setting during mealtime. Children were assessed using the DDS while consuming liquids, non-chewable solid foods (puree), and chewable solids that they had brought from home. Families were also asked to bring any assistive utensils the children used during meals. During the evaluation, children were instructed to take at least five sips or bites of each liquid or food item [14]. The natural feeding situation was neither altered nor interrupted by the test leader for the benefit of the study. Some children were even assessed in positions close to a supine posture due to their physical needs. To minimize excitement or fear, especially among dystonic children, the caregiver was present during the evaluation alongside the evaluator and the child. However, it was observed that some children displayed heightened excitement and increased dystonic movements during the in-person assessment, which appeared to hinder their ability to demonstrate their true swallowing performance. To address this limitation, caregivers were instructed as part of a second method to record videos of their children during mealtime in their home environment. These videos, captured using smartphone cameras, followed the DDS protocol for standardized food and liquid consumption. The video recordings were then submitted to the research team for evaluation. The same evaluator reviewed the videos, and discrepancies between the clinical and video-based assessments were analyzed and detailed in the Results section. Finally, dysphagia severity was determined using the Dysphagia Management Staging Scale (DMSS), a severity scale developed in conjunction with the DDS [14].

### Measurements

The descriptive characteristics of the children, including their age, sex, diagnosis, predominant movement disorder, and current medications, were recorded. Additionally, the number of pneumonia episodes experienced within the past year, potentially related to swallowing dysfunction, was documented.

#### Gross motor function level.

Gross motor function level was assessed using the Gross Motor Function Classification System (GMFCS), which classifies motor abilities into five levels, ranging from Level I (most independent) to Level V (most dependent) [15].

#### Oral intake level.

Oral intake levels were determined using the Functional Oral Intake Scale (FOIS), a reliable and valid tool for assessing functional oral intake. The FOIS includes seven levels, where Level 1 represents “nothing by mouth,” and Level 7 represents “total oral diet with no restrictions.” [16].

#### Oral structure and hygiene assessment.

A comprehensive oral structure assessment was conducted by a certified swallowing therapist (M.N.K.), which included observing the presence or absence of open mouth posture, open bite, tongue thrust, high-arched palate, and oral hygiene [17]. Additionally, the presence of trismus was recorded as either present or absent. Oral hygiene was assessed by counting the number of missing, filled, or decayed teeth and noting the presence of halitosis and tooth brushing habits (recorded as present if brushing occurred at least once daily).

#### Chewing.

Chewing was evaluated using two methods. First, the Turkish version of Mastication Observation and Evaluation (T-MOE), a reliable and valid tool that assesses tongue protrusion, lateral tongue movement, squashing or sucking movements, jaw movement, chewing duration, food or saliva loss, number of swallows, and fluency/coordination [18]. Each item was scored on a 4-point ordinal scale, with lower scores reflecting poorer performance and higher scores indicating better performance [19]. Second, the Karaduman Chewing Performance Scale (KCPS) was used to classify chewing performance into five levels, ranging from Level 0 (normal chewing function) to Level IV (inability to bite or chew) [20]. To ensure consistency, standardized biscuits were used during the assessments, and the children’s chewing performance was observed while consuming the biscuits.

#### Drooling severity and frequency.

The Drooling Severity and Frequency Scale was used to determine the severity and frequency of drooling in children [21]. This scale evaluates the severity and frequency of drooling based on observation or information provided by parents or caregivers. Drooling severity is scored on a scale from 1 to 5, where level 1 indicates no drooling and dry lips, level 2 indicates mild drooling, level 3 represents moderate drooling, level 4 corresponds to severe drooling, and level 5 signifies excessive drooling that wets clothing. Drooling frequency is scored on a scale from 1 to 4, where level 1 indicates no drooling, level 2 represents rare drooling, level 3 indicates frequent drooling, and level 4 corresponds to constant drooling [21].

#### Tongue thrust.

The Tongue Thrust Rating Scale (TTRS), which is a valid, reliable, clinically easy to use, was used to determine tongue thrust severity in children [22]. The scale defines the severity of tongue thrust in four levels, ranging from 0 to 3. Level 0 indicates ‘No tongue thrust’, Level 1 indicates ‘Mild tongue thrust’ Level 2 indicates ‘Moderate tongue thrust’ and Level 3 indicates ‘Severe tongue thrust’.

#### Swallowing safety assessment.

Swallowing safety was assessed using the 3-ounce water swallow test. Each child was asked to drink 3 ounces of water from a cup without interruption while their natural position (e.g., sitting in their personal wheelchair or supported seating), to reflect daily life conditions. If the patient is unable to hold the cup, the clinician assists them. Referral for further assessment was warranted if the child exhibited coughing, choking, or wet/hoarse vocal quality during or within one minute of completing the test. Performance was scored as “successful” if no signs of impaired safety were observed and “failed” if any of the criteria were met [23,24]. In addition, the PEDI-EAT-10 questionnaire, a parent-proxy tool, was administered to assess penetration and aspiration risks [25]. This 10-item questionnaire, scored on a 5-point ordinal scale, indicated greater risk of unsafe swallowing with higher scores. A score of ≥4 [25] indicated swallowing dysfunction, and a score of ≥13 [26] indicated a heightened risk of aspiration.

#### Swallowing disorder severity.

Swallowing disorder severity was determined using the DDS, a validated tool for children aged two years and older [14]. The DDS, which has demonstrated strong internal consistency and validity, includes 15 items divided into two sections: Part 1 assesses related factors such as body mass index, diet, independence, postural control, diet consistency, adaptive utensils, special feeding techniques, and seating alignment, while Part 2 evaluates feeding and swallowing competency, including orienting, food reception, containment, oral transport, chewing, oral-pharyngeal swallowing, post-swallow, and esophageal swallowing. In line with the developers’ recommendations, items 1 and 15 were excluded from the scoring due to weak associations with other items, resulting in total scores ranging from 0 to 34. Higher scores indicated greater swallowing disorder severity [27]. The DMSS was used to classify the DDS scores into five levels of severity: Level 1 (no disorder), Level 2 (mild disorder), Level 3 (moderate disorder), Level 4 (severe disorder), and Level 5 (profound disorder) [14].

### Ethical approval

Ethical approval for the study was obtained from the Gazi University Ethics Committee (Protocol number: 2023/1107) and written informed consent was taken from all participants. The research was conducted by the ethical guidelines as outlined in the Declaration of Helsinki. The individual in the supplementary video has given written informed consent (as outlined in the PLOS consent form) to publish these case details.

### Statistical analysis

Statistical analyses were performed using SPSS version 25.0 (SPSS Inc., Chicago, IL, USA). Normal distribution for continuous variables was assessed with visual (histograms and probability graphics) and the Shapiro-Wilk test. Descriptive statistics for categorical variables were presented as frequencies and percentages, while continuous variables with normal distribution were reported as mean ± standard deviation (SD). Dependent sample t-tests were used to compare DDS raw sore clinic and DDS raw score home. Statistical significance was set at *p* < 0.05 (two-tailed).

## Results

A total of 25 children were included in the study, with 56.0% being female. The mean age of the participants was 11.32 ± 3.95 years. Among the dystonic children, 9 were diagnosed with CP, 3 with pantothenate kinase-associated neurodegeneration (*PKAN*), 3 with *KMT2B*, 2 with *SLC18A2*-related dystonia, 2 with *WARS2*, and 2 with suspected neurotransmitter disorder. Additionally, 56% of the children were classified as level V according to the GMFCS. Furthermore, three children (12%) had a FOIS level of 4 or below. Detailed demographic and clinical characteristics of the children are summarized in Table 1

**Table 1:** Demographics and Clinical Characteristics of Children with Dystonia (n:25).

Child	Age (years)	Sex	Diagnosis	Predominant Movement Disorder	Current Medications	Surgery History	Pneumonia Episodes (last 1 year)	GMFCS level (I-V)	FOIS level (1-7)
**1**	8	M	GNAO1	Dystonia+ Chorea	Tetrabenazin, Gabapentin, L-Dopa, Vigabatrin, Clobazam		2	V	5
**2**	16	F	PKAN	Dystonia	Trihexyphenidyl, Baclofen		2	V	4
**3**	13	F	PKAN	Dystonia	Clonazepam, Haloperidol, Baclofen, Clonidine, Gabapentin, Risperidone	Pallidotomy	2	V	2
**4**	16	F	PKAN	Dystonia	Baclofen, Trihexyphenidyl		2	V	3
**5**	14	M	KMT2B	Dystonia	Trihexyphenidyl, L-Dopa	DBS	–	II	7
**6**	11	M	KMT2B	Dystonia	Trihexyphenidyl, Gabapentin, Essitalopram		–	II	7
**7**	13	M	KMT2B	Dystonia	Trihexyphenidyl, L-Dopa, Risperidone, Sertraline, Guanfacine		–	II	7
**8**	11	M	CP	Dystonia	L-Dopa, Gabapentin, Trihexyphenidyl		–	II	7
**9**	17	M	CP	Dystonia	Baclofen, Gabapentin		–	IV	7
**10**	17	F	CP	Dystonia	–		–	IV	7
**11**	16	M	CP	Dystonia	Baclofen, L-Dopa, Clonezepam, Trihexyphenidyl	DBS	2	V	6
**12**	16	M	CP	Dystonia	Baclofen, Trihexyphenidyl Tetrabenazine		–	II	7
**13**	12	F	CP	Dystonia+ Chorea	Baclofen		–	V	7
**14**	10	F	CP	Dystonia	Clonezepam, Trihexyphenidyl Gabapentin, Pyridoxine		1	V	6
**15**	10	F	CP	Dystonia+ Chorea	Clonezepam, Primidone		–	III	7
**16**	13	M	CP	Dystonia	Baclofen, Tizanidine		–	III	7
**17**	5	M	SLC18A2	Dystonia+Parkinsonism	Clobazam, Valproate		1	V	5
**18**	5	F	SLC18A2	Dystonia+Parkinsonism	Gapapentin, Pramipexole		2	V	5
**19**	11	F	Mitochondrial Disease	Dystonia	–		2	V	6
**20**	5	F	Suspected Neurotransmitter Disease	Dystonia+Parkinsonism	Gabapentin, L-Dopa		2	V	5
**21**	5	F	Suspected Neurotransmitter Disease	Dystonia+Parkinsonism	Gabapentin, L-Dopa		2	V	5
**22**	8	M	WARS2	Dystonia	–		–	V	6
**23**	13	F	WARS2	Dystonia	–		1	V	5
**24**	17	F	HSP15/ZFYVE26	Dystonia	–		–	II	7
**25**	11	F	PRKRA	Dystonia+Parkinsonism	L-dopa		–	IV	6

M: Male; F: Female; DBS: Deep Brain Stimulation; GMFCS: Gross Motor Function Classification System, Level I (least impaired) to V (most impaired); FOIS: Functional Oral Intake Scale, Level 1 (nothing by mouth) to 7 (total oral intake with no restrictions).

In terms of oral structure characteristics, 84% of children with dystonia presented with halitosis, 64% had decayed teeth, 56% exhibited tongue thrust, and 16% were identified with trismus. The comprehensive oral structure characteristics of the children are displayed in Table 2.

**Table 2:** Oral Structure Characteristics of Children with Dystonia (n:25).

	n (%)
**Open Mouth**	8 (32)
**Open Bite**	8 (32)
**Tongue Thrust**	14 (56)
**High-Arched Palate**	7 (28)
**Missing Teeth**	10 (40)
**Filled Teeth**	14 (56)
**Decayed Teeth**	16 (64)
**Halitosis**	21 (84)
**Tooth Brushing**	4 (16)
**Trismus**	4 (16)

**Fig 1:**
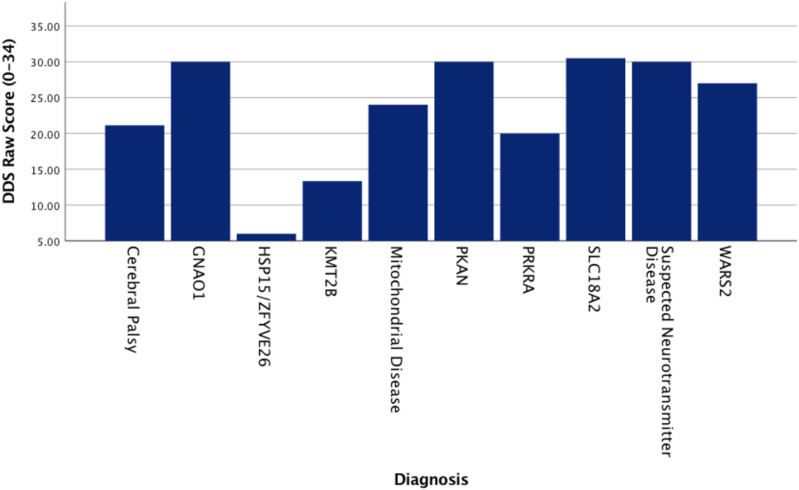
DDS raw score distribution by diagnosis. Scale.

The mean T-MOE score among the children was 15.62 ± 7.51. Additionally, 60% of the children had a KCPS level of 3 or above, while 52% had a drooling severity level of 3 or higher. Regarding swallowing performance, 52.0% of the children failed the 3-ounce water swallow test. All participants had a PEDI-EAT-10 score of 4 or above, and 88% had a score of 13 or higher, reflecting substantial challenges in swallowing and feeding. The mean DDS raw score was 23.08 ± 7.70, with 68% of the children classified as having severe or profound dysphagia based on the DMSS. Children with *PKAN* and *SLC18A2*-related dystonia had higher DDS raw scores compared to those with *KMT2B*-related dystonia, as illustrated in Fig 1. Clinical swallowing characteristics of the children with dystonia are provided in Table 3.

**Table 3:** Clinical Swallowing Characteristics of Children with Dystonia (n:25).

Child	T-MOE score (0-32)	KCPS (0–4)	Drooling Severity Scale (1-5)	Drooling Frequency Scale (1–4)	Tongue Thrust Rating Scale (0–3)	3-ounce Water Swallow Test	PEDI-EAT-10 (0–40)	DDS Raw Score (0–34)	DMSS level
**1**	11	3	4	3	2	Failed	25	30	Profound
**2**	*	*	3	3	0	*	40	30	Profound
**3**	*	*	2	2	0	*	38	33	Profound
**4**	*	*	4	3	0	*	32	30	Profound
**5**	18	1	2	2	0	Successful	21	19	Severe
**6**	25	1	1	1	0	Successful	6	5	Mild
**7**	26	1	1	1	0	Successful	5	16	Moderate
**8**	17	2	2	2	1	Successful	15	20	Severe
**9**	22	2	3	3	0	Failed	32	23	Severe
**10**	17	3	3	3	1	Failed	24	21	Severe
**11**	20	3	3	3	2	Failed	25	25	Severe
**12**	24	2	1	1	0	Successful	17	13	Moderate
**13**	20	3	2	2	0	Failed	28	23	Severe
**14**	18	3	3	3	2	Failed	33	27	Severe
**15**	21	1	2	2	0	Successful	19	17	Moderate
**16**	20	3	2	2	1	Successful	22	19	Severe
**17**	14	3	3	3	2	Failed	36	31	Severe
**18**	12	3	3	2	2	Failed	33	30	Severe
**19**	12	3	3	3	1	Failed	24	24	Severe
**20**	9	3	4	3	2	Failed	25	30	Severe
**21**	9	3	4	4	2	Failed	27	30	Severe
**22**	15	2	2	2	2	Failed	18	22	Moderate
**23**	13	3	3	3	2	Failed	31	32	Profound
**24**	27	0	1	1	0	Successful	7	6	Mild
**25**	24	2	2	2	1	Successful	24	20	Severe

* Test not performed; T-MOE: Turkish Version of the Mastication and Observation Evaluation; KCPS: Karaduman Chewing Performance Scale, PEDI-EAT-10: The Pediatric Version of the Eating Assessment Tool-10; DDS: Dysphagia Disorder Survey, DMSS: Dysphagia Management Staging Scale.

There was no significant difference between DDS scores obtained in clinical and home settings (23.08 ± 7.70 vs. 22.83 ± 7.47, p > 0.05), reflecting similar levels of swallowing impairment in both environments.

## Discussion

This study is the first to thoroughly investigate the clinical swallowing characteristics of children with dystonia. The results demonstrated impaired swallowing function in all participants, with severe dysfunction observed in more than half of the cases. Significant findings included pathological reflexes and drooling during the oral phase, poor oral hygiene, and inadequate chewing function. Additionally, over half of the children presented an increased risk of aspiration. While these results provide an overview of the key findings, further analysis revealed that the etiological diagnosis underlying dystonia significantly influenced clinical swallowing characteristics.

A detailed medical history was obtained for each child, with a particular focus on pneumonia episodes within the past year. This approach was informed by literature suggesting an association between recurrent pneumonia and swallowing disorders, particularly in children with neurological impairments [28–30]. In our study, 12 children (48.0%) had experienced at least one episode of pneumonia in the previous year. Children diagnosed with *PKAN*, suspected neurotransmitter disease, *SLC18A2*, or *GNAO1* were particularly noteworthy, as all had recurrent pneumonia. Further examination of these children revealed common clinical features: all had a GMFCS level of V, varying degrees of chewing dysfunction and drooling, failed the 3-ounce water swallow test, and were classified as having severe or profound dysphagia according to the DMSS scale. These findings are clinically significant. Despite evident impairments in swallowing safety based on clinical evaluations, only two children were receiving tube feeding. Both of these children were diagnosed with *PKAN* and were tube-fed due to severe trismus rather than recurrent respiratory infections. The remaining 10 children continued oral feeding, although their clinical evaluations and medical histories raised concerns about the safety of oral intake. This highlights the need for instrumental swallowing assessments, such as videofluoroscopic swallowing study (VFSS) or fiberoptic endoscopic evaluation of swallowing (FEES), to confirm clinical findings. However, motor impairments associated with dystonia often make these assessments challenging. Stabilizing these children during VFSS or FEES is frequently impractical, limiting the feasibility of such evaluations. Additionally, some families resist transitioning their children to non-oral feeding despite medical recommendations, choosing instead to continue oral feeding. Additionally, some families resist transitioning their children to non-oral feeding despite medical recommendations, choosing instead to continue oral feeding. Based on our clinical experience, this resistance often stems from the belief that oral feeding represents the child’s connection to life and a vital caregiving act. In line with this, recent research has shown that caregivers may experience significant emotional and cognitive challenges during the decision-making process regarding gastrostomy insertion. In a study by Kinsella et al. [31], only 64.8% of caregivers reported always feeling included in decision-making, while others reported feeling only sometimes (19.7%) or almost never (8.5%) involved. Furthermore, 27.8% stated that the tube should have been inserted earlier, suggesting retrospective regret and a possible lack of clarity in communication. These findings highlight that for many families, the feeding tube decision is not merely a clinical intervention, but one deeply entwined with emotional, relational, and cultural factors. These challenges emphasize the need for regular and comprehensive swallowing assessments in children with dystonia, particularly in those with specific etiological diagnoses.

When examining the oral structures of children with dystonia, our findings aligned with those reported in studies on children with CP [10,32]. One of the most prevalent pathological oral reflexes observed was tongue thrust. Tongue thrust is associated with prolonged sucking behavior beyond infancy. In our study, tongue thrust was identified in 60% of children with dystonia. This reflex is also commonly observed in children with CP and has been linked to structural abnormalities such as open mouth posture, open bite, and high-arched palate [33,34]. Tongue thrust significantly affects oral phase function, particularly chewing, which requires multidirectional tongue movements. Children with tongue thrust tend to compress food using anterior-posterior tongue movements rather than proper chewing mechanics. This reflex is also strongly associated with drooling, as reported in previous studies [33,34]. Our findings are consistent with the literature; all children with tongue thrust demonstrated varying degrees of chewing dysfunction and drooling.

Another notable finding was poor oral hygiene. The oral cavity serves as a reservoir for bacteria that can lead to various systemic diseases, including respiratory illnesses. Studies have demonstrated that the number of decayed and missing teeth is significantly associated with the incidence of pneumonia [35,36]. Research on children with CP has shown that factors such as drooling, dental caries, oral motor dysfunction, and residual food, combined with the absence of regular tooth brushing, negatively impact oral hygiene and increase the risk of pneumonia [37]. In our study, nearly half of the children had missing or decayed teeth, and almost all exhibited halitosis. Given the established relationship between poor oral hygiene and pneumonia risk in CP, further research focusing on oral hygiene interventions in children with dystonia is warranted.

A unique clinical observation in children with dystonia was trismus, which was present in all three children diagnosed with *PKAN*. Trismus, characterized by an inability to fully open the jaw, posed a significant challenge [38]. Two of these children were tube-fed due to severe trismus, which had led to persistent injuries and bleeding in the oral cavity, including damage to the gums, tongue, and lips. Their inability to brush their teeth further worsened their poor oral hygiene. These findings highlight the need for targeted interventions to address trismus in children with rare disorders such as *PKAN*.

Regarding clinical swallowing assessments, all children in the study exhibited varying levels of chewing dysfunction and drooling. Chewing function could not be assessed in children with *PKAN* due to trismus. However, children with CP and those diagnosed with *KMT2B* exhibited relatively better chewing function. Another noteworthy finding about these children was the use of sensory tricks by some children with cervical dystonia. Sensory tricks involve tactile stimulation to temporarily reduce dystonic symptoms. For example, some children would touch their faces or move their heads toward their shoulders to stabilize themselves during swallowing.

From a swallowing safety perspective, a substantial portion of the children, (88% scored ≥13 on the PEDI-EAT-10), were found to be at high risk for unsafe swallowing. While this finding aligns with reports of recurrent pneumonia, a rare clinical observation emerged: a few children who were fed in a supine position-typically considered unsafe-yet had no documented history of pneumonia. This was surprising, as their clinical evaluations indicated impaired swallowing safety. During initially assessments, we requested home videos from families to observe feeding practices, expecting that feeding in a supine position would significantly increase aspiration risk. However, despite prolonged feeding in this position, these children had not experienced pneumonia in the past one or two years. Unlike findings in children with spastic CP, where improved posture enhances swallowing safety, adjusting the posture of children with dystonia often compromised their ability to swallow [39]. For instance, one child began coughing on liquids when placed in an upright position. These findings underscore the importance of individualized assessments that incorporate both clinical evaluations and medical history. Children with dystonia may present unique postural and motor characteristics, highlighting the need for regular, personalized follow-ups in swallowing evaluation. Given the high aspiration risk identified in this study, early identification and structured screening for dysphagia should be a priority in pediatric dystonia care. Validated tools such as the DDS and the 3-ounce water swallow test can be easily implemented in outpatient settings. Furthermore, individualized interventions including postural adjustments, feeding modifications, and caregiver training should be integrated into routine multidisciplinary management. These approaches may help reduce aspiration-related complications and foster family engagement in care.

In this study, the DDS assessments of children with dystonia were compared using home videos and clinical evaluations. We decided to do this because, during the initial assessments, we noticed increased motor disturbances and anxiety while the children were eating in the clinic. Families also mentioned that their children ate better at home. For this reason, we evaluated the children in both settings. Although some children showed slightly better performance at home, the results were similar across both environments. We believe this similarity was due to maintaining a calm clinical atmosphere and giving the children enough time to adjust to the environment and the evaluators.

This study is the first to provide an in-depth examination of swallowing function in children with dystonia. Over a year, 25 children with various etiological backgrounds were included. The severity of their swallowing disorders was assessed using validated clinical tools such as the DDS and the DMSS. Additionally, different DDS scoring methods were analyzed to account for the impact of motor impairments in various settings. These aspects represent the strengths of our study. However, several limitations should be noted. First, the DDS assessments were conducted by a single certified therapist, which precluded the evaluation of interrater reliability. Second, the potential influence of medications should be considered. Several participants were receiving drugs such as trihexyphenidyl, clobazam, risperidone, sertraline, guanfacine, L-dopa, and valproate, which may affect swallowing through mechanisms including reduced salivation, altered muscle tone, sedation, or impaired motor coordination. Due to the heterogeneity in medication types and dosing regimens, these effects could not be systematically analyzed. Third, the heterogeneity of etiological diagnoses in the sample may limit the generalizability of the findings to populations with more homogeneous clinical profiles. Finally, the small sample size and the inability to perform instrumental assessments such as VFSS or FEES due to motor instability posed methodological challenges. These factors highlight the need for future studies with larger, more etiologically uniform samples and additional instrumental evaluations to verify the findings.

## Conclusion

This study uncovers a pervasive and severe swallowing dysfunction in children with dystonia, highlighting the complexity of their condition and the significant clinical challenges it presents. Key findings include significant oral motor dysfunctions, including pathological reflexes, drooling, and impaired chewing, all of which compromise swallowing safety and increase the risk of aspiration. Despite these risks, many children continue oral feeding, making it critical to provide urgent, tailored, multidisciplinary care to prevent life-threatening complications. Routine swallowing assessments in clinical settings are essential for these children to ensure early detection and appropriate intervention of any further complications.

The variability in swallowing dysfunctions based on the underlying etiology of dystonia underscores the need for individualized care, particularly for children with different clinical profiles. Longitudinal studies are essential to monitor the progression of swallowing impairments and assess the effectiveness of interventions such as oral hygiene practices and adaptive feeding techniques. These studies will be crucial for refining management strategies and improving the overall quality of life for children with dystonia.
